# The SDF1-CXCR4 Axis Is Involved in the Hyperbaric Oxygen Therapy-Mediated Neuronal Cells Migration in Transient Brain Ischemic Rats

**DOI:** 10.3390/ijms23031780

**Published:** 2022-02-04

**Authors:** Ray-Yau Wang, Yea-Ru Yang, Heng-Chih Chang

**Affiliations:** 1 Department of Physical Therapy and Assistive Technology, National Yang Ming Chiao Tung University, Taipei 112, Taiwan; rywang@nycu.edu.tw (R.-Y.W.); yryang@nycu.edu.tw (Y.-R.Y.); 2 Department of Physical Therapy, Asia University, Taichung 413, Taiwan

**Keywords:** brain ischemia, hyperbaric oxygen therapy, neurogenesis, stromal cell-derived factor-1, CXC chemokine receptor 4

## Abstract

Neurogenesis is a physiological response after cerebral ischemic injury to possibly repair the damaged neural network. Therefore, promoting neurogenesis is very important for functional recovery after cerebral ischemic injury. Our previous research indicated that hyperbaric oxygen therapy (HBOT) exerted neuroprotective effects, such as reducing cerebral infarction volume. The purposes of this study were to further explore the effects of HBOT on the neurogenesis and the expressions of cell migration factors, including the stromal cell-derived factor 1 (SDF1) and its target receptor, the CXC chemokine receptor 4 (CXCR4). Thirty-two Sprague–Dawley rats were divided into the control or HBO group after receiving transient middle cerebral artery occlusion (MCAO). HBOT began to intervene 24 h after MCAO under the pressure of 3 atmospheres for one hour per day for 21 days. Rats in the control group were placed in the same acrylic box without HBOT during the experiment. After the final intervention, half of the rats in each group were cardio-perfused with ice-cold saline followed by 4% paraformaldehyde under anesthesia. The brains were removed, dehydrated and cut into serial 20μm coronal sections for immunofluorescence staining to detect the markers of newborn cell (BrdU^+^), mature neuron cell (NeuN^+^), SDF1, and CXCR4. The affected motor cortex of the other half rats in each group was separated under anesthesia and used to detect the expressions of brain-derived neurotrophic factor (BDNF), SDF1, and CXCR4. Motor function was tested by a ladder-climbing test before and after the experiment. HBOT significantly enhanced neurogenesis in the penumbra area and promoted the expressions of SDF1 and CXCR4. The numbers of BrdU^+^/SDF1^+^, BrdU^+^/CXCR4^+^, and BrdU^+^/NeuN^+^ cells and BDNF concentrations in the penumbra were all significantly increased in the HBO group when compared with the control group. The motor functions were improved in both groups, but there was a significant difference between groups in the post-test. Our results indicated that HBOT for 21 days enhanced neurogenesis and promoted cell migration toward the penumbra area in transient brain ischemic rats. HBOT also increased BDNF expression, which might further promote the reconstructions of the impaired neural networks and restore motor function.

## 1. Introduction

Stroke is a major cause of death and disability worldwide. Cerebral ischemic injury is the most common form of stroke. The field of the middle cerebral artery is the most common involved area in brain ischemic injury. The impaired areas of the brain after ischemic insult include the ischemic core and penumbra area [1]. The cells in the ischemic core area are necrotic and non-reversible. However, for the cells in the penumbra area, cell apoptosis is reduced if appropriate therapeutic interventions are given within a critical time limit. Therefore, early intervention to reduce cerebral ischemic injury has always been an important guideline for the clinical treatment of stroke patients.

Neurogenesis is a normal physiological phenomenon after brain ischemic injury. There are two main areas in the brain that have the function to regenerate new progenitor cells, one is the subventricular zone (SVZ), and the other is the subgranular zone of the dentate gyrus in the hippocampus [2,3,4]. The new progenitor cells produced in the SVZ can transmit along the rostral migratory stream to the olfactory bulb and migrate to the sites of injury [2,5]. Therefore, enhancing the progenitor cells migration to the lesion sites has the potential to help the nervous system to self-repair and may help patients to rebuild their function after cerebral ischemic injury.

It has been demonstrated that stromal cell-derived factor 1 (SDF1) and its target receptor, C-X-C motif chemokine receptor 4 (CXCR4) controls the migration of neural progenitor cells [6]. The expressions of SDF1 and CXCR4 are upregulated in the ischemic brain [7,8]. SDF1-CXCR4 axis has also been suggested to promote the survival and migration of transplanted bone marrow stromal cells toward the lesion site [9,10] and regulate the inflammatory responses and focal angiogenesis [8] in brain ischemic rats. In the traumatic brain injury rat model, the SDF1-CXCR4 axis also promotes the migration of endogenous neural stem cells (NSCs) [11]. Cui and colleagues indicated the SDF1-CXCR4 axis plays a particularly important role in adult neurogenesis, including mediating the proliferation and migration of neural progenitor cells [12]. Therefore, regulating the SDF1-CXCR4 signaling might provide in maximizing the amount of migrated NSCs in the penumbra area and contribute to functional recovery after stroke.

Since the last decades, there have been many studies exploring the therapeutic benefits of hyperbaric oxygen therapy (HBOT) on brain ischemia. HBOT refers to providing 100% oxygen above one atmospheric pressure in a pressure control chamber for a specific period of time. HBOT has been demonstrated to reduce cell apoptosis, blood-brain barrier damage, cerebral edema, inflammation, intracranial pressure, lipid peroxidation, and free radical formation, stimulate vasculogenic stem cell growth, and improve energy metabolism in brain ischemic rats [13,14,15,16,17,18,19,20,21,22]. In recent years, HBOT has been suggested to play a role in promoting the proliferation of neural progenitor cells within the SVZ in neonatal rats with hypoxic-ischemic brain injury, and in adult rats with brain ischemic injury and traumatic brain injury [23,24,25,26,27]. These studies indicate the intervention of HBO after brain ischemia may be effective to protect neural cells from damage and promote neural plasticity. However, it is not fully known about the involvements of SDF1-CXCR4 axis in the HBOT. The purposes of the present study were aimed to explore the effects of HBOT for 21 days on the protein expressions of SDF1 and CXCR4 and the migration of newborn cells in the penumbra area in rats with transient middle cerebral artery occlusion (MCAO) injury.

## 2. Results

### 2.1. HBO Improves the Motor Function

**Figure 1:**
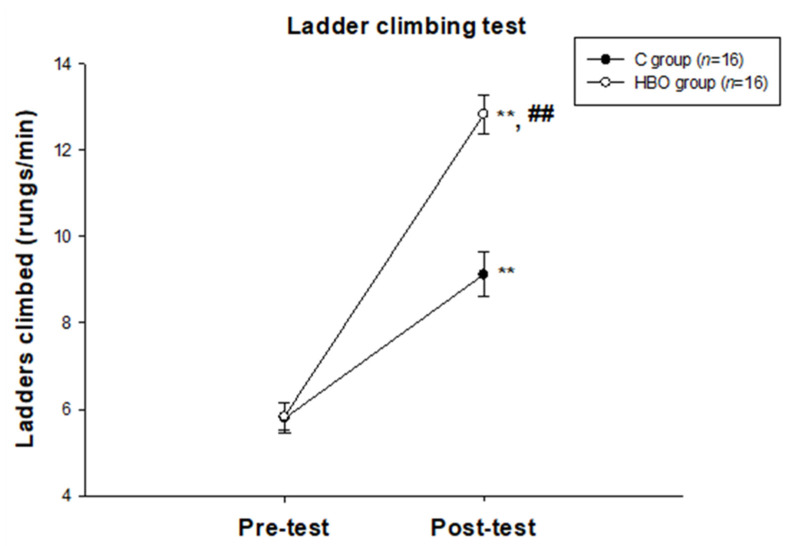
The results of ladder climbing test between groups. **, *p* < 0.01 compared to the pre-test data. ##, *p* < 0.01 compared to the C group. C: control; HBO: hyperbaric oxygen.

The result of the ladder-climbing test is shown in Figure 1. There was no significant difference between groups in the pre-test. The motor functions in the post-test (C group: 9.12 ± 0.52; HBO group: 12.83 ± 0.44) were significantly improved in both groups when compared with the pre-test (C group: 5.79 ± 0.36, *p* < 0.01; HBO group: 5.83 ± 0.31, *p* < 0.01). The motor function in the post-test was shown a significant difference between groups (*p* < 0.01). The results indicated that HBO intervention significantly enhanced motor function recovery.

### 2.2. HBOT Enhances SDF1 and CXCR4 Expressions in the Affected Motor Cortex

**Figure 2:**
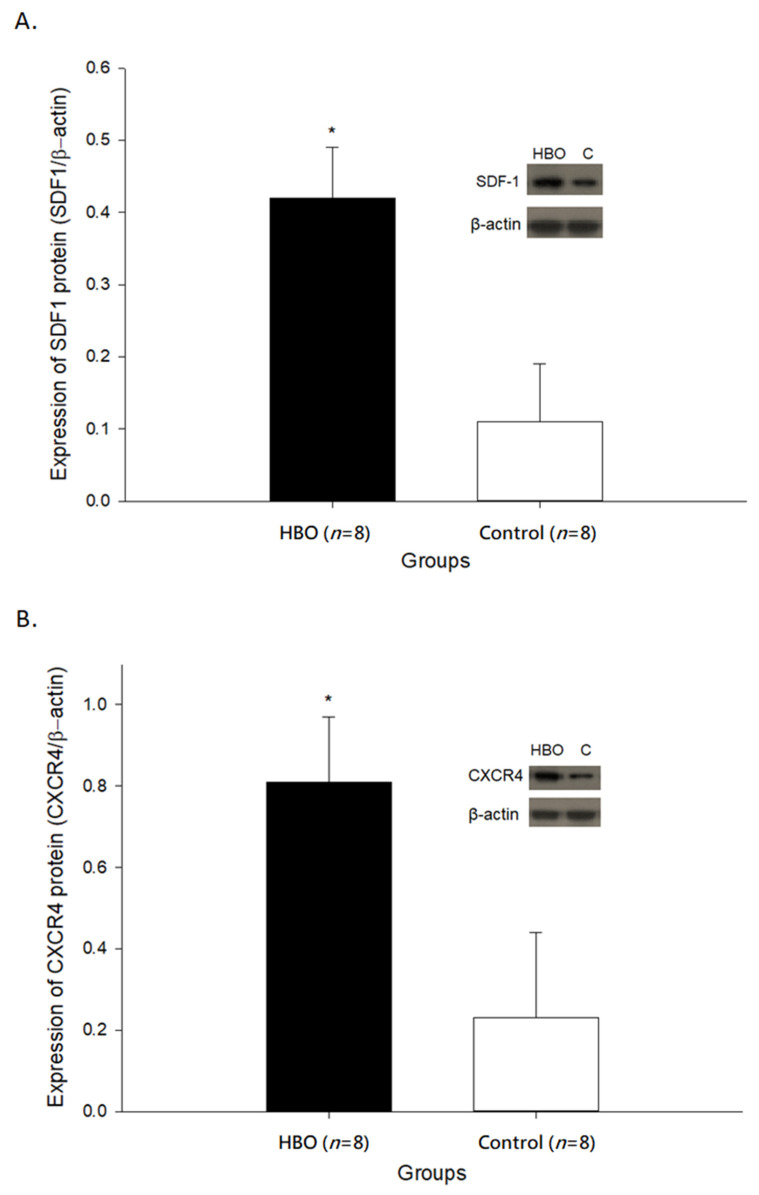
(**A**) The expressions of SDF1 in the affected motor cortex between groups. (**B**) The expressions of CXCR4 in the affected motor cortex between groups. *, *p* < 0.05. C: control; HBO: hyperbaric oxygen; SDF1: Stromal cell-derived factor 1; CXCR4: CXC chemokine receptor 4.

The expressions of SDF1 and CXCR4 in the affected motor cortex are shown in Figure 2. The protein expressions of SDF1 (0.42 ± 0.07) and CXCR4 (0.81 ± 0.16) in the HBO group were both significantly increased when compared with the control group (SDF1: 0.11 ± 0.08, *p* < 0.05; CXCR4: 0.23 ± 0.21, *p* < 0.05). These results indicated that HBOT enhanced the expressions of cell migration factors, including the SDF1 and its targeted receptor CXCR4 in the affected motor cortex.

### 2.3. HBOT Enhances Neurogenesis and Cell Migration

**Figure 3:**
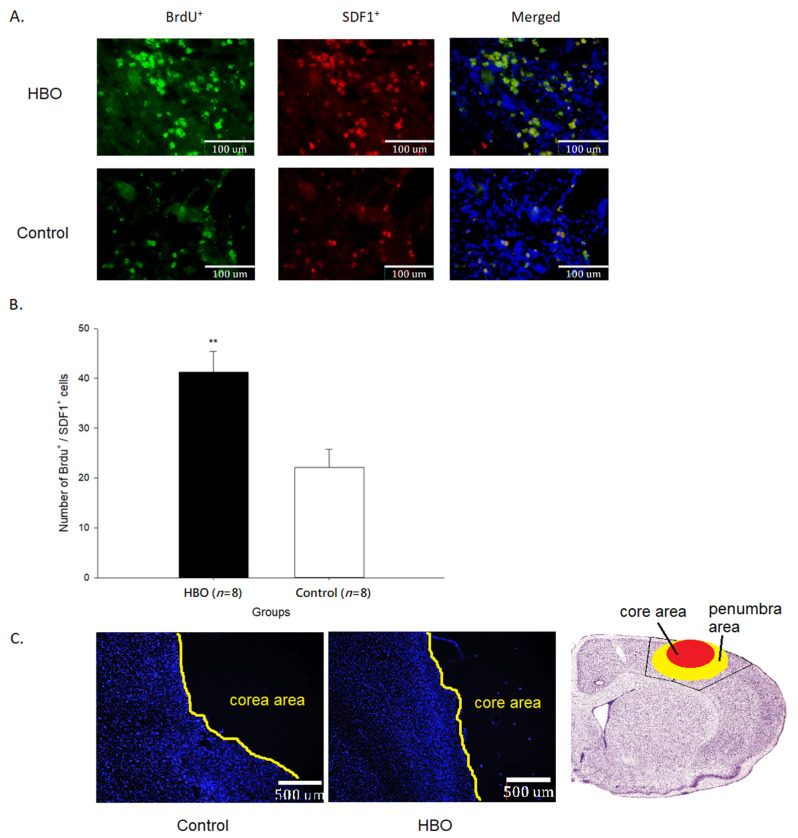
(**A**) The immunofluorescence imaging of BrdU^+^ (green) and SDF1^+^ (red) cells in the penumbra area between groups (under 40× objective lens). (**B**) The quantifications of BrdU^+^/SDF1^+^ cells in the penumbra area between groups. (**C**) Illustration of penumbra and core area boundaries (under 5× objective lens). Nuclei stained with DAPI (blue). **, *p* < 0.01. HBO: hyperbaric oxygen; SDF1: Stromal cell-derived factor 1.

**Figure 4:**
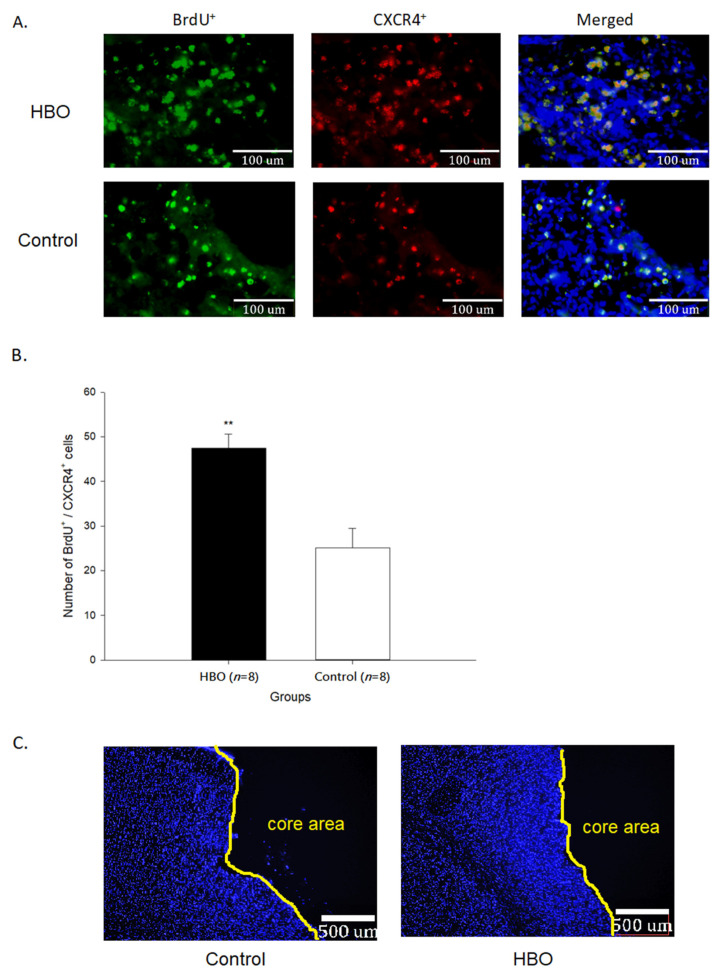
(**A**) The immunofluorescence imaging of BrdU^+^ (green) and CXCR4^+^ (red) cells in the penumbra area between groups (under 40× objective lens). (**B**) The quantifications of BrdU^+^/CXCR4^+^ cells in the penumbra area between groups. (**C**) Illustration of penumbra and core area boundaries (under 5× objective lens). Nuclei stained with DAPI (blue). **, *p* < 0.01. HBO: hyperbaric oxygen; CXCR4: CXC chemokine receptor 4.

The results of immunofluorescence of BrdU^+^ and SDF1^+^ cells in the penumbra area are shown in Figure 3. The newborn cells (BrdU^+^) in the penumbra area of the HBO group were much more than that in the control group. The quantifications of BrdU^+^/SDF1^+^ cells (41.28 ± 4.13 cells) were significantly increased in the HBO group when compared with the control group (22.18 ± 3.64 cells, *p* < 0.01). The results of immunofluorescence of BrdU^+^ and CXCR4^+^ cells in the penumbra area are shown in Figure 4. The quantifications of BrdU^+^/CXCR4^+^ cells (47.41 ± 3.18 cells) were significantly increased in the HBO group when compared with the control group (25.08 ± 4.34 cells, *p* < 0.01). These results indicated that HBOT promoted the newborn cells migrating toward the penumbra area through the SDF1-CXCR4 axis.

### 2.4. HBOT Promotes Differentiation of Neurons

**Figure 5:**
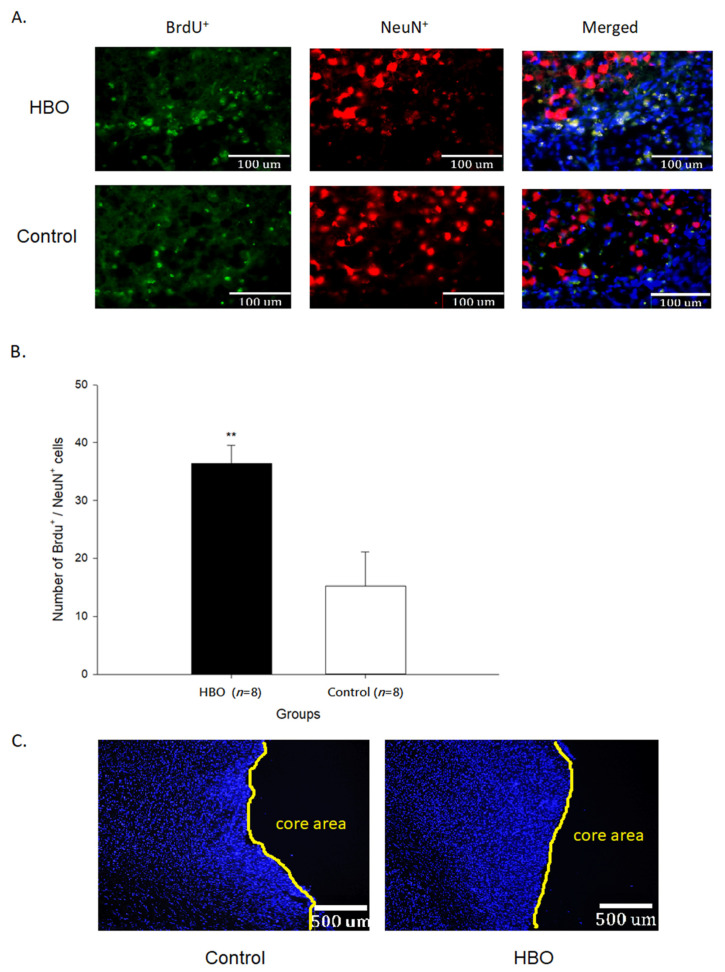
(**A**) The immunofluorescence imaging of BrdU^+^ (green) and NeuN^+^ (red) cells in the penumbra area between groups (under 40× objective lens). (**B**) The quantifications of BrdU^+^/NeuN^+^ cells in the penumbra area between groups. (**C**) Illustration of penumbra and core area boundaries (under 5× objective lens). Nuclei stained with DAPI (blue). **, *p* < 0.01. HBO: hyperbaric oxygen.

The results of immunofluorescence of BrdU^+^ and NeuN^+^ cells in the penumbra area are shown in Figure 5. The quantifications of BrdU^+^/NeuN^+^ cells (36.42 ± 3.08 cells) were significantly increased in the HBO group when compared with the control group (15.21 ± 5.86 cells, *p* < 0.01). These results indicated that the migratory progenitor cells were partially differentiated into the matured neurons and HBOT enhanced the differentiative properties.

### 2.5. HBOT Up-Regulates BDNF Expression in the Affected Motor Cortex and Serum

**Figure 6:**
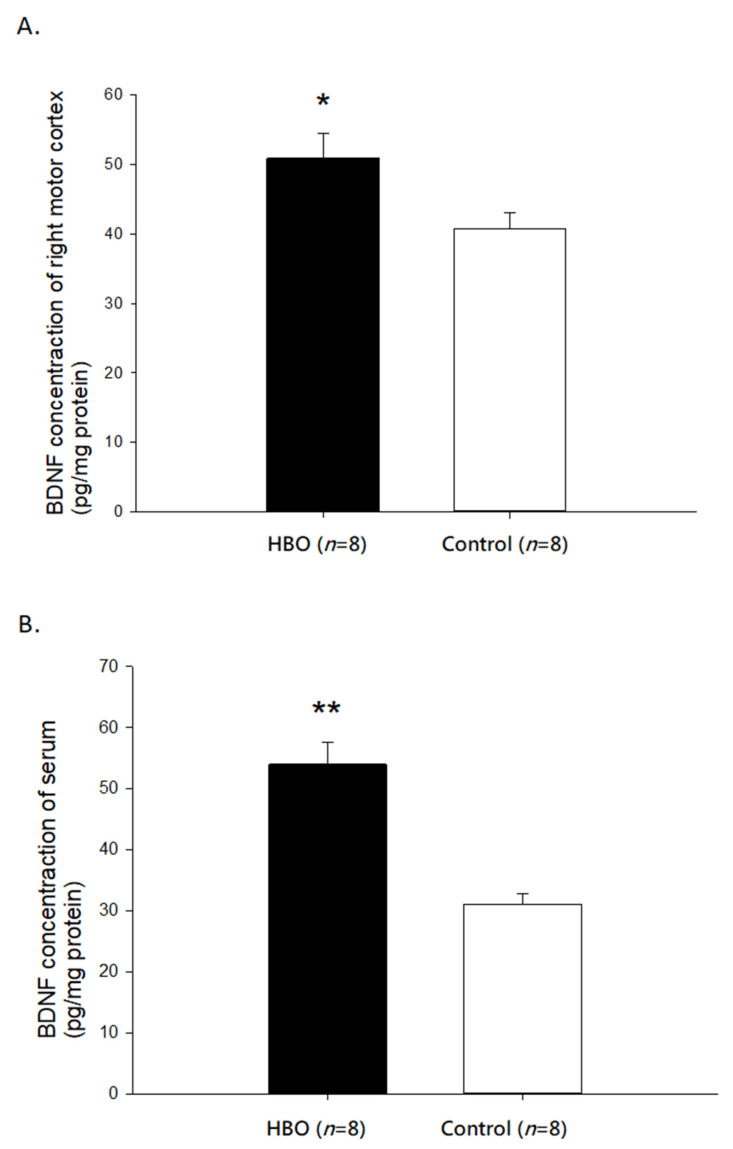
(**A**) The expression of BDNF in the affected motor cortex between groups. (**B**) The expression of BDNF in the serum between groups. *, *p* < 0.05; **, *p <* 0.01. HBO: hyperbaric oxygen; BDNF: Brain-derived neurotrophic factor.

The expression of BDNF in the affected motor cortex and serum is shown in Figure 6. HBOT significantly increased the expression of BDNF in the affected motor cortex (50.87 ± 3.66 pg/mg protein) when compared with the control group (40.66 ± 2.34 pg/mg protein, *p* < 0.05). The serum BDNF level in the HBO group (54.04 ± 3.59 pg/mg protein) was also significantly increased than that in the control group (31.10 ± 1.78 pg/mg protein, *p* < 0.01). These results indicated that HBOT could increase the expressions of neurotropic factors, such as BDNF.

## 3. Discussion

Neurogenesis is a natural mechanism of the nervous system, which continues throughout life to provide the plasticity of the nervous system. Many studies confirmed that damage to the nervous system will promote neurogenesis [28,29,30]. Several factors regulate neurogenesis including the fibroblast growth factor 2, epidermal growth factor, pigment epithelium-derived factor, betacellulin, vascular endothelial growth factor, glial cell line-derived neurotrophic factor, nerve growth factor, and BDNF et al. [4,31,32]. Inhibition of neurogenesis can result in poor functional recovery and synaptic connection in the ischemic brain [33,34]. In addition, Penti and colleagues indicated that there is a peak increase in cell proliferation in the SVZ on the 14th day, followed by a subsequent decrease on the 28th day post-MCAO [35]. Therefore, it is important to promote neurogenesis as soon as possible after the brain suffers from ischemic injury.

In our previous study, HBOT for 14 to 21 days provides a significant reduction in the infarct volume and improvement in motor function [21]. In the present study, HBOT for 21 days significantly increased the expressions of SDF1 and CXCR4 and neurogenesis in the penumbra area. Therefore, based on the results of these studies, we may suggest that the sustained HBOT after cerebral ischemic insult not only exerts a neuroprotective effect but also promotes the newborn cells migrating toward the penumbra area.

In addition to being able to migrate to damaged brain regions, the neural progenitor cells must also be able to differentiate into mature cells and integrate into the neural network, so that they can repair the damaged nervous system effectively. In the present study, we noted that some of the newborn cells in the penumbra area differentiated into the mature neuron cells, especially in the HBO group. This result could be inferred that sustained HBOT might also promote the differentiation of new neuronal cells. Ardelt and colleagues reported that application of SDF1 after brain ischemia-reperfusion injury modulates synaptic transmission to the neural progenitors in the peri-lesion site. They concluded that SDF1 plays a role in regulating neurogenesis during the repair process after brain ischemia [36]. It has also been suggested that SDF1-CXCR4 axis promotes neural progenitor cells differentiation and neuronal cells survival [37,38]. In the present study, the expressions of SDF1 and CXCR4 in the penumbra area were both significantly increased after HBOT. Besides, BDNF concentration in the penumbra area and serum were also significantly increased in the HBO group. It is well-known that BDNF not only protects neural cells from injury but also plays an important role in neuroplasticity [39,40]. Meng and colleagues demonstrated HBOT promotes significant functional recovery by activating the SDF1-CXCR4 axis and increases the expression of BDNF in the incomplete spinal cord injury rat model [41]. Taken together, modulations of SDF1-CXCR4 axis and BDNF expression by HBOT as shown in the present study might participate in the repair process after brain ischemia and contribute to motor function improvement.

A recent study report that the SDF1 and CXCR4 axis may be a possible prognostic indicator of clinical acute ischemic stroke. The plasma levels of SDF1-α and the numbers of circulating CD34^+^/CXCR4^+^ cells measured within seven days show negative correlations to the prognostic values measured by the modified Rankin scale on the 90-day post-ischemia. This result indicates that increases in the expressions of SDF1-CXCR4 axis during the acute phase of ischemic stroke are associated with better functional recovery of daily activity [42]. Other clinical studies indicated that HBOT reduces functional impairments and improves neurocognitive functions in stroke patients [43,44,45]. Therefore, the utilization of HBO intervention in clinical trials might be a potential strategy for patients with brain ischemia.

The underlying mechanisms of HBOT in modulating neuroplasticity after brain ischemia are still not fully known. It has been noted that the HBOT increases the expression of hypoxia-inducible factor 1α (HIF-1α) in brain ischemic rats [26]. HIF-1α is mainly a gene regulatory factor stimulated by hypoxia, which can regulate at least 100 genes related to promoting cell survival [46]. One possible mechanism for the increase in HIF-1α after HBOT may be the change of oxygen level mimicking the hypoxia-like condition. The other proposed mechanism may be HBOT modulates the ratio of reactive oxygen species (ROS) and ROS scavengers. HIF-1α is quickly hydroxylated when ROS interacts with the HIF-1 hydroxylated proteins in the normoxic state. However, repeated HBOT up-regulate the contents of ROS scavengers and prolong their half-life. Therefore, if more ROS is eliminated by HBOT, more HIF-1α can be reserved and enter the nucleus to dimerize with the HIF-1β to form the active HIF promoter. [47]. Our previous study also confirmed that HBOT enhances the antioxidative effects in brain ischemic rats [21]. Furthermore, HIF-1α is suggested to promote neurogenesis in brain ischemic rats and regulate the SDF1-CXCR4 axis to enhance bone marrow-derived mesenchymal stromal cell migration in rats with traumatic brain injury [26,48]. Therefore, the up-regulation of the SDF1-CXCR4 axis as shown in the present study might also be related to the effect of HBO on the modulation of HIF-1α. Future experiments are suggested to confirm this inference.

Finally, although the results of the present study indicated that sustained HBOT promoted the migration of newborn cells toward the penumbra area through the SDF1-CXCR4 axis, there were some limitations that should be addressed. First, the types of newborn cells besides the neuronal cells were not investigated. Second, the interactions of cells were not investigated, such as the synaptic connections and signal transduction among cells et al. Future studies are suggested to investigate these points to better understand the effects of HBOT after brain ischemia.

## 4. Materials and Methods

### 4.1. Animals and Grouping

32 male Sprague-Dawley rats (8 weeks of age, body weight 300–350 g) were randomly divided into the normal air control (C, *n* = 16) or hyperbaric oxygen (HBO, *n* = 16) groups after receiving transient middle cerebral artery occlusion (MCAO). Animals in the HBO group were given HBOT at a pressure of 3 atmospheres for one hour, starting from 24-h post MCAO, once a day for 21 days. Animals in the control group were given a relative rest intervention for 21 days. All experiments were performed during daytime. Animals were housed in an environment with an automatic light cycle (light on between 7:00 A.M. and 7:00 P.M) and constant temperature control (22 ± 1 °C) and provide unlimited food and drinking water. All experimental procedures had been reviewed and approved by the Institutional Animal Care and Use Committee (IACUC) of National Yang-Ming Chiao Tung University, Taiwan, R.O.C. (IACUC number: 1090609).

### 4.2. Middle Cerebral Artery Occlusion

Animals received transient MCAO surgery under sodium pentobarbital anesthesia (50 mg/kg BW) as described in our previous studies [21,49]. In brief, A 2 mm burr hole was drilled at the junction of the squamous bone and the right zygomatic arch. The right middle cerebral artery (MCA) trunk was explored and occluded by a 10-0 suture needle. The blood flow was completely interrupted and confirmed under a microscope. According to the original model descriptions, bilateral common carotid arteries (CCAs) were occluded using nontraumatic aneurysm clips to successfully reduce the blood flow in the supplied field of MCA [50]. After 1 h of occlusion, the aneurysm clips and the 10-0 suture needle were removed. The blood flow in all three arteries was observed directly under a microscope to ensure fully retorted. During the MCAO procedures, the rectal temperature was monitored and maintained at 37.0 ± 0.5 °C by a temperature-controlled heating blanket (WATLOW 050100C1, Bowdoinham, ME, USA). Rats were returned to their cages after the wound was sutured and fully recovered from anesthesia. 24 h post-MCAO, the neurological score was tested according to the neurological grading scale (0–4) [51]. Median neurological scores were 3 (range: 1–3) in the normal air control group and 3 (range: 1–3) in the HBO group, respectively. The neurological scores did not differ significantly between groups.

### 4.3. Hyperbaric Oxygen Therapy

The HBOT was administered in an acrylic glass pressure chamber (UO 300AR, United Oxygen Biotech, Inc., Taipei, Taiwan). 24 h post-MCAO, rats in the HBO group received HBOT at a pressure of 3 atmospheres without air breaks for 1 h with 100% oxygen, once a day for 21 days. To ensure the oxygen level, an oxygen sensor (MAXO2 Oxygen Sensor, Maxtec, Salt Lake City, UT, USA) was used during HBOT, and the oxygen level was maintained at 100% O_2_ [21,52]. In order to absorb the accumulation of CO_2_ during HBOT, a box of calcium carbonate crystals was placed at the corner of the chamber and renewed every day [53]. The compression and decompression were achieved within 5 min before and after the 1 h HBO intervention. Rats in the C group were placed in the same box as used in the HBO group and sham-treated in the pressure chamber for 1 h, once a day for 21 days.

### 4.4. Motor Behavior Test

Motor-behavior performance was tested at two time points, including 24 h post-MCAO (pre-test) and 2 h post the final experimental intervention (post-test). The ladder-climbing test was used to determine the coordination of all four limbs as described in previous studies [49,54]. In brief, rats were encouraged to climb a stainless-steel vertical ladder (2 cm interval) for 1 min and the number of rungs climbed within 1 min was recorded. The test was performed 3 times with a 5 min rest, and the average score was used for comparison. These tests were performed by a well-trained research assistant who was blinded to group allocation.

### 4.5. Tracking the Neurogenesis Cells

In order to trace the newborn cells in the penumbra area, rats were intraperitoneally injected with Bromodeoxyuridine (5-Bromo-2′-deoxyuridine, BrdU, 50 mg/kg body weight) before the daily experimental interventions started from 24 h post-MCAO. BrdU was dissolved in sterile PBS solution (pH = 6.8).

### 4.6. Sample Preparation

After the final motor behavior test, half of the rats in each group were anesthetized with an overdose of anesthetic (sodium pentobarbital, 100 mg/kg), and cardio-perfused with 40-mL ice-cold PBS (pH = 6.8) and 60-mL ice-cold 4% paraformaldehyde/PBS (pH = 6.8). The brain was removed and dehydrated with 30% sucrose/PBS (pH = 6.8), and then cut into slices with a thickness of 20 μm each on a cryostat (CM3050S, Leica, Buffalo Grove, IL, USA) from 1.5 mm anteriorly to −0.5 mm posteriorly of the Bregma, which involves the motor and somatosensory areas of the rat brain [55]. The brain slices were stored at −20 °C before the immunofluorescent exanimation.

The other half of the rats in each group were anesthetized with an overdose of anesthetic (sodium pentobarbital, 100 mg/kg), the brain was quickly removed and rinsed with ice-cold PBS (pH = 6.8) to remove excess blood. The right motor cortex was collected and ground with the lysis buffer (SI-C3228, Sigma-Aldrich, St. Louis, MI, USA) containing cocktail protease inhibitors (ROC-04693132001, Sigma-Aldrich, St. Louis, MI, USA). The tissue lysates were centrifuged at a speed of 12,500-rpm for 30 min at 4 °C, then the tissue supernatants were separated for the subsequent analysis. The total protein concentration in the supernatant was measured with a Bradford-red protein detection reagent (SI-B6916, Sigma-Aldrich, St. Louis, MI, USA).

### 4.7. Immunofluorescent Examination

The procedures of immunofluorescence staining are according to the previous study with a minor modification [2]. The brain slices were fixed by immersing in the acetone for 10 min and rehydrated in the PBS for 10 min at room temperature. Brain slices were then incubated in the 2N HCl for 30 min at 37 °C and 10 nM sodium citrate buffer (pH = 6.0) for 5 min at 85 °C to break down the DNA chain and retrieve the antigens. After washing in the PBS for 5 min, the brain slices were incubated in an immunofluorescence blocking buffer (12411S, Cell Signaling, Danvers, MA, USA) for 1 h at room temperature and washed in the PBS for 5 min thrice. Brain slices were then incubated in the antibody dilution buffers (12378S, Cell Signaling, Danvers, MA, USA) containing the mixtures of rat anti-BrdU-Alexa Fluor 488 (1:500, ab220074, Abcam, Cambridge, UK) and rabbit anti-NeuN-Alexa Fluor 674 (1:500, ab190565, Abcam, Cambridge, UK) antibodies or rat anti-BrdU-Alexa Fluor 488 and rabbit anti-CXCR4 (1:250, ab124824, Abcam, Cambridge, UK) antibodies or rat anti-BrdU-Alexa Fluor 488 and rabbit anti-SDF1 (1:500, PA5-114344, Invitrogen, Waltham, MA, USA) antibodies overnight at 4 °C. After washing the slices in the PBS for 5 min thrice (protect from light), the brain slices were then incubated in the antibody dilution buffer containing the goat anti-rabbit IgG Alexa Fluor 647 antibody (1:1000, A21244, Invitrogen, Waltham, MA, USA) in the dark for 1 h at room temperature, excepted for the slices that were incubated in the mixture containing rat anti-BrdU-Alexa Fluor 488 and rabbit anti-NeuN-Alexa Fluor 674 antibodies. The slices were washed in the PBS for 5 min thrice, mounted with an antifade solution with DAPI (8961, Cell Signaling, Danvers, MA, USA), and sealed by cover-glass (protect from light). The fluorescence emitted was observed through each appropriate filter on fluorescence microscopy (Leica DM 6000B, Leica, Wetzlar, Germany) and was digitally photographed using a cooled CCD camera. Three non-overlapping areas within the penumbra area of each brain slice were photographed for calculating the target cells of interest. Six brain slices per rat were used in the present study.

### 4.8. Brain-Derived Neurotrophic Factor Examination

10 μL of tissue supernatant and serum for each sample was taken and used to determine the concentration of BDNF by using an ELISA kit (BEK-2211, Biosensis, Thebarton SA, Australia). The procedures were according to the manufacturer’s protocols. The concentration of BDNF was presented as pg/mg protein in the present study.

### 4.9. SDF1 and CXCR4 Protein Examination

For western blot, an equal amount of protein (30 mg) from each sample was resolved using a 12% SDS-PAGE and the proteins on the colloid were transferred onto the polyvinylidene fluoride membrane (IPVH00010, Millipore, Burlington, MA, USA). The membrane was incubated in the 0.1% Tween 20/tris-buffered saline (0.1% TBST, pH = 8.0) containing 5% skimmed milk to fill and block the non-specific bonding sites on the membrane for 1 h at room temperature. After washing the membrane with 0.1% TBST for 5 min thrice, the membrane was incubated in the 0.05% TBST (pH = 8.0) containing 3% skimmed milk and primary antibodies for the specific proteins at 4 °C overnight. The excess antibodies that were not bound to the target protein on the membrane were washed out with 0.1% TBST thrice (10 min each wash) and then incubated in the 0.05% TBST containing 3% skimmed milk and horseradish peroxidase-conjugated secondary antibodies for 1 h at room temperature. After another three-times of wash with 0.1% TBST, the membrane was incubated in the western blot chemiluminescence reagent (XR-IGE-RPN2106, Sigma-Aldrich, St. Louis, MI, USA) for 3 min, and the expression of the target protein was detected with X-ray film. The specific protein primary antibodies used in the present study include the rabbit anti-SDF1 (1:1000, PA5-114344, Invitrogen, Waltham, MA, USA), rabbit anti-CXCR4 (1:1000, ab124824, Abcam, UK) and mouse anti-β-actin (1:3000, MAB8929, R&D system, Minneapolis, MN, USA); The secondary antibodies include the goat anti-rabbit HRP (1:6000, ab205718, Abcam, Cambridge, UK) and goat anti-mouse HRP (1:6000, ab205719, Abcam, Cambridge, UK). The signals of protein expression were detected and quantified using an Image-Gauge software (Fujifilm, Minato-ku, Tokyo, Japan). The β-actin was used as the standard protein. The expressions of SDF1 and CXCR4 were presented as a ratio to β-actin.

### 4.10. Statistical Analysis

All data were expressed as mean ± standard error of mean. An independent t-test was performed to determine the differences in the numbers of BrdU^+^/NeuN^+^, BrdU^+^/SDF1^+^, and BrdU^+^/CXCR4^+^ cells and the protein expressions of BDNF, SDF1, and CXCR4 between groups. Differences in motor tests were examined by two-way repeated-measures (group *x* time) ANOVA. Significance was set at *p* < 0.05.

## Data Availability

The data that support the findings of this study are available from the corresponding author upon reasonable request.
